# Does mobile phone ownership predict better utilization of maternal and newborn health services? a cross-sectional study in Timor-Leste

**DOI:** 10.1186/s12884-016-0981-1

**Published:** 2016-07-23

**Authors:** Juan Nie, Jennifer Anna Unger, Susan Thompson, Marisa Hofstee, Jing Gu, Mary Anne Mercer

**Affiliations:** School of Public Health, Sun Yat-sen University, No. 74 Zhongshan 2nd Road, Guangzhou, 510080 People’s Republic of China; Sun Yat-sen Global Health Institute, Sun Yat-sen University, No. 74 Zhongshan 2nd Road, Guangzhou, 510080 People’s Republic of China; Department of Global Health, University of Washington, 1510 N.E. San Juan Road, Seattle, WA 98195-7965 USA; Department of Obstetrics & Gynecology, University of Washington, Harborview Medical Center, 325 Ninth Ave, Box 359865, Seattle, WA 98104 USA; Health Alliance International, 1107 NE 45th Street, Suite 350, Seattle, WA 98105 USA

**Keywords:** mHealth, Mobile phone ownership, Maternal, newborn, and child health, Health services

## Abstract

**Background:**

Increasingly popular mobile health (mHealth) programs have been proposed to promote better utilization of maternal, newborn and child health services. However, women who lack access to a mobile phone are often left out of both mHealth programs and research. In this study, we determine whether household mobile phone ownership is an independent predictor of utilization of maternal and newborn health services in Timor-Leste.

**Methods:**

The study included 581 women aged 15–49 years with a child under the age of two years from the districts of Manufahi and Ainaro in Timor-Leste. Participants were interviewed via a structured survey of knowledge, practices, and coverage of maternal and child health services, with additional questions related to ownership and utilization of mobile phones. Mobile phone ownership was the exposure variable, and the dependent variables included having at least four antenatal care visits, skilled birth attendance, health facility delivery, a postnatal checkup within 24 h, and a neonatal checkup within 24 h for their youngest child. Logistic regression models were applied to assess for associations.

**Results:**

Sixty-seven percent of women reported having at least one mobile phone in the family. Women who had a mobile phone were significantly more likely to be of higher socioeconomic status and to utilize maternal and newborn health services. However, after adjusting socioeconomic factors, household mobile phone ownership was not independently associated with any of the dependent variables.

**Conclusion:**

Evaluations of the effects of mHealth programs on health in a population need to consider the likelihood of socioeconomic differentials indicated by mobile phone ownership.

**Electronic supplementary material:**

The online version of this article (doi:10.1186/s12884-016-0981-1) contains supplementary material, which is available to authorized users.

## Background

Maternal, newborn, and child health (MNCH) has recently attracted increased global attention. United Nations Millennium Development Goal (MDG) 4 focused on reducing the mortality of children under 5, while MDG 5 advocated global involvement in reducing the maternal mortality ratio [1, 2]. The subsequent Sustainable Development Goal for health targeted specific reductions in maternal, newborn and child health [3]. Although recent evidence indicates steady progress is being made towards these goals, maternal and neonatal mortality remain global challenges [4]. It is estimated that almost 300,000 women died of pregnancy, delivery, or postnatal complications in 2013 worldwide, with 99 % of these deaths occurring in low to middle income countries (LMIC) [5]. Annually there are approximately 3 million neonatal deaths, the majority of which occur in LMICs [6]. Although there are many factors contributing to these deaths, inadequate utilization of maternal and newborn health services remains a barrier to reducing maternal morbidity and improving newborn health. In 2012, nearly 40 million births were not attended by skilled birth personnel and only fifty-two percent of pregnant women received the recommended minimum of four antenatal care visits in developing regions [5]. Thus, additional strategies are needed to promote better utilization of health services related to pregnancy and childbirth.

The penetration of mobile phone services has provided health workers and health systems access to populations that have been difficult to reach by traditional approaches [7]. According to the International Telecommunications Union, the number of mobile cellular subscriptions will reach seven billion globally by the year of 2014, with more than three quarters of those in the developing world [8]. Mobile health (mHealth), which represents a variety of health programs utilizing mobile phones, has been increasingly applied in LMIC countries to achieve better health outcomes.

With the emergence of mHealth programs, health workers have seized opportunities to utilize mobile phones to connect health systems with pregnant women. Text messaging, as a low cost, accessible, and convenient tool for health education, medical reminders, and communication, is playing a role in promoting mutual interaction between women of reproductive age and health care providers [9]. mHealth via text messaging has also been a means of delivering health information and direct health services [10]. Recent evaluations of mHealth programs have demonstrated that various forms of text messaging can increase the utilization of antenatal care, skilled birth attendance and postnatal health services while improving customer satisfaction [11–16].

Despite the rapid development and early benefits of mHealth programs, most of these interventions focus on the population who already have mobile phones, leaving non- mobile phone owners without similar support and investigation. Although mHealth interventions have been shown to reach people in rural and remote areas [17], inequities in mobile phone access still remain [18–21]. Effective program development requires an understanding of populations without access to a mobile phone, including their health care utilization. In this study, we aim to investigate socioeconomic factors and the utilization of maternal and newborn health services among a population of postpartum women with and without access to mobile phones in rural Timor-Leste. We also explore the association between access to mobile phones and the utilization of health services related to pregnancy and childbirth among women in this setting.

## Methods

### Study setting

According to the 2009/2010 Demographic and Health Survey (DHS) [22], Timor-Leste has one of the highest Maternal Mortality Ratios (MMR) in the world, 557 deaths per 100,000 live births. Despite 86 % attendance by pregnant women for at least one antenatal care visit (ANC1^+^), only 55 % complete the recommended four ANC visits (ANC4^+^) during pregnancy [22]. Approximately 30 % of women utilize skilled birth attendants (SBA) for their delivery, and one in five births are delivered in a health facility. The majority of women (68 %) do not receive any postnatal check [22]. Mobile phones are increasingly popular among women in Timor-Leste, where in 2009 only 40 % of households owned a mobile phone [22], and by 2012 in Manufahi and Ainaro districts, where our study took place, 69 and 66 % of women respectively had a household mobile phone.

### Study design

A baseline survey was conducted in February and March, 2012, in Ainaro and Manufahi districts prior to the launch of the *Liga Inan* mobile phone project of Health Alliance International (HAI) to assess the level of knowledge, practices, and health service coverage related to maternal health, and to collect information about mobile phone ownership and usage patterns in both districts. This study is a secondary data analysis of data from that baseline survey.

### Data sources

Institutional Review Board approval for the study was obtained from the University of Washington Human Subjects Division, as well as from the Cabinet for Health Research and Development, an institution overseeing ethical review of all health research in Timor-Leste. Approval for a waiver of written consent was obtained because a number of women were expected to be unable to sign their names. Interviewers approached eligible women to explain the survey and ask them if they would participate, and then documented their informed consent prior to the interview (please see Additional file 1). If participants under the age of 17 were unmarried, consent from a parent/guardian was obtained. If participants under the age of 17 were married, consents were provided by themselves, as married girls between 15 or 16 were considered emancipated according to the Timor-Leste Civil Code.

The survey questionnaire was developed from the USAID’s Rapid CATCH (Core Assessment Tool on Child Health) Survey [23], combined with questions retrieved from a Demographic and Health survey (DHS) that was conducted in Timor-Leste [24]. Additional questions about mobile phone use were developed by HAI project staff and tested extensively in the field. Data were collected on demographic characteristics; number and timing of antenatal care visits, birthing practices, postnatal care, and neonatal care; and ownership and utilization of mobile phones. The English questionnaire was translated into the local language of Tetun, and interviews were conducted in Tetun.

### Participants

Women aged 15–49 years old with a child up to 24 months of age were eligible for the baseline survey, conducted in February and March 2012 in Ainaro and Manufahi districts. The survey employed stratified cluster sampling. In each of the 8 total subdistricts, 8 clusters of 9 households, totaling 72 households per subdistrict, were included using a standard formula for cluster survey sampling [25]. If there were two women in the household, the mother with the youngest child was interviewed. If women who met the selection criteria were absent from the house, the study team would return if possible. Sixteen eligible women were excluded from the study because they were not at home and not expected to return. If the original selected village did not contain enough participants, researchers found substitute participants from the nearest village. Seventy-two participants were enrolled in each subdistrict except for the subdistrict of Turiscai, which had 77 participants. In total, 581 women were interviewed.

### Variables

Socio-demographic characteristics, mobile phone ownership, and selected maternal and newborn health services were characterized using descriptive statistics. We included demographic characteristics (age, years of schooling, household commodities, materials of the roof and floor, literacy), number of births, number of children, and travel time to the nearest health facility in the analysis. Household ownership of mobile phones was the exposure variable of interest. We coded our exposure variable as *mobile phone owners* and *non-mobile phone owners,* defined as women who did or did not have access to a mobile phone in their household. Outcome variables included *ANC4+* (defined as had antenatal care visits four or more times during last pregnancy), *SBA* (had a skilled birth attendant, such as a midwife or a doctor, deliver their last baby*), health facility delivery* (delivered their last baby at a health facility)*, early postnatal care* (had a postnatal checkup by a health worker within 24 h after delivery either at a health facility, home or other location)*, and early neonatal care* (had their newborn baby checked by a health worker within 24 h after the baby was born)*.*

Original continuous data such as age, years of school, number of children, and travel time to the nearest health facility were classified into categories. Based on the methods introduced in DHS [26–29], we calculated the wealth index by compositely measuring the cumulative living standards based on household assets: electricity, radio, television, bicycle, car or truck, horse or other animal powered transport, type of roofing, and type of flooring. Principal Component Analysis (PCA) was applied to assess the factor weight of each asset. The wealth index, which is a continuous variable, was then divided into five indices, which are Highest, Fourth, Middle, Second, and Lowest levels [29].

### Statistical methods

Demographic characteristics of women with a mobile phone and those who did not have a mobile phone were described by the frequency and percentage, and were compared using the chi-square test for categorical data. The chi-square test was also used in bivariate analysis to explore the relationship of independent variables, including mobile phone ownership, with utilization of maternal and newborn health services. We included independent variables that could be potential confounders to the outcomes into the logistic model, including the covariates of age, education, parity, wealth index, travel time to the nearest health facility, and literacy. Variables are selected stepwise by backward elimination from the original list, with variables that have *p* values less than 0.20 removed from the model. Odds ratios (OR) and 95 % confidence intervals (CI) are reported.

Data entry and sample size calculation were completed with Epi Info 7.0. We used STATA version 12.0 (StataCorp, Texas) to conduct data analysis, and set 0.05 as the significance level (alpha). The *svyset STATA* command was used to adjust cluster effects of our data collection. We weighted the results of the survey to accommodate for the difference in the population size or sample size between clusters, subdistricts, and the two program districts.

## Results

### Participants

A total of 581 participants from Manufahi and Ainaro districts were enrolled in the study. Three hundred and sixty four (67 %) participant households owned at least one mobile phone. Baseline characteristics of participants are shown in Table 1. The mean age of participants was 27.8 years [SD = 0.39, 95 % CI: 27.0–28.6]. About half of women (52 %) had received secondary or above education while two-thirds were capable of reading and/or writing. The majority of the women (70 %) lived within one hour of the nearest health facility, and half (49 %) had at least four children. Less than half the women, 46 %, attended their first visit before the gestational age of 3 months, and 95 % before 6 months.

**Table 1:** Demographic characteristics of study participants

Variables	N (%) of the participants			*P* ^***^
	Mobile phone owners 67 % (*N* = 364)	Non mobile phone owners 33 % (*N* = 217)	Total (*N* = 581)	
Age				0.240
≤20	47(13)	30(14)	77(13)	
21–24	69(21)	46(19)	115(20)	
25–29	121(32)	48(24)	169(29)	
30–34	66(18)	41(20)	107(19)	
≥35	61(16)	52(24)	113(19)	
Mean (year) (SD)	27.5(0.44)	28.3(0.66)	27.8(0.39)	
Education level				0.000
No	51(16)	75(39)	126(23)	
Primary	84(22)	65(28)	149(24)	
Secondary or above	229(62)	77(33)	306(52)	
Wealth Index				0.000
Lowest	45(12)	73(33)	118(19)	
Fourth	52(12)	68(34)	120(19)	
Middle	74(22)	45(20)	119(22)	
Second	92(26)	15 (6)	107(19)	
Highest	101(28)	16(6)	117(21)	
Parity				0.360
1	57(15)	33(14)	90(15)	
2–3	128(38)	74(32)	202(36)	
≥4	179(46)	110(53)	289(49)	
Health facility travel time^a^				0.060
0–60 min	261(74)	139(62)	400(70)	
>60 min	103(26)	77(38)	180(30)	
Literacy				0.000
Cannot read and write	81(25)	105(54)	186(34)	
Can read or write	283(75)	112(46)	395(66)	
District				0.640
Mahufahi	172(51)	121(48)	293(50)	
Ainaro	192(49)	96(52)	288(50)	
Gestational months at 1st ANC visit^b^	345	188	533	0.090
1–3	175(48)	94(42)	269(46)	
4–6	162(49)	82(50)	244(49)	
7–9	8(3)	12(8)	20(5)	

^a^One data point is missing

^b^533 women had at least 1 ANC visit when they had their last child

^***^All *p*-values are produced using Chi-square test, adjusted for cluster effect

### Demographics and mobile phone ownership

Table 1 compares the demographic characteristics of participants according to household ownership of mobile phones. In comparison to participants without mobile phones, those with mobile phones were significantly more likely to be educated (*p* = 0.000), wealthy (*p* = 0.000), and more capable of reading and/or writing (*p* = 0.000). Women with mobile phones were also more likely to be located closer to health facilities, with a relationship of borderline significance (*p* = 0.060),

### Utilization of maternal and newborn health services and mobile phone ownership

We examined the utilization of maternal and newborn health services among survey participants: ANC 4+, SBA, health facility delivery, and postnatal and neonatal care within 24 h of delivery. A majority of the participants (72 %) achieved ANC 4+. Less than half (43 %) of participants reported SBA at their last delivery, and only one-third (31 %) delivered their last baby at a health facility. Approximately one-third (31 %) of women reported receiving a postnatal checkup by a health worker within 24 h of the delivery, and 34 % reported having a postnatal checkup after 24 h of the delivery. A quarter reported bringing their baby in for a neonatal checkup within 24 h of delivery, and 39 % more than 24 h after delivery.

The unadjusted utilization rates for women who had a household mobile phone as compared to those without a phone were significantly higher for ANC 4+ (OR = 1.92; 95 % CI: 1.23–3.00; *p* = 0.005), delivering with a skilled birth attendant (OR = 2.45; 95 % CI: 1.55–3.86; *p* = 0.000), having health facility delivery (OR = 2.68; 95 % CI: 1.61–4.44; *p* = 0.000), and receiving a postnatal checkup within 24 h after delivery (OR = 2.11; 95 % CI: 1.43–3.14; *p =* 0.000) and neonatal checkup within 24 h after delivery (OR = 1.74; 95 % CI: 1.05–2.90; *p =* 0.032) (Table 2).

**Table 2:** Association between mobile phone ownership and health services related to pregnancy and childbirth

Variable	Mobile phone owners (*N* = 364)	Non mobile phone owners (*N* = 217)	Unadjusted OR 95 % CI	*P*	Adjusted OR 95 % CI^a^	*P* ^**^
Antenatal care				0.005		0.133
ANC ≥4	264(76)	136(63)	1.92(1.23,3.00)		0.74(0.51,1.09)	
ANC <4	100(24)	81(37)	Ref.		Ref.	
Skilled birth attendance				0.000		0.100
Yes	174(50)	61(29)	2.45(1.55,3.86)		1.29(0.67,2.51)	
No	190(50)	156(71)	Ref.		Ref.	
Health Facility Delivery				0.000		0.115
Yes	113(37)	33(18)	2.68(1.61,4.44)		1.13(0.56,2.28)	
No	251(63)	184(82)	Ref.		Ref.	
PNC within 24 h				0.000		—
Yes	128(36)	45(21)	2.11(1.43,3.14)		—	—
No	236(64)	172(79)	Ref.		Ref.	—
NNC within 24 h				0.032		—
Yes	100(29)	140(19)	1.74(1.05,2.90)		—	
No	264(71)	177(81)	Ref.		Ref.	

^a^Adjusted for cluster effect, and factors related to the outcomes. Factors include age, education, wealth index, parity, travel time to the nearest health facility, and literacy

^**^All *p*-value are produced using the maximum likelihood ratio test

The multivariate model for mobile phone ownership included parity as well as the socioeconomic variables for age of mother, education level, wealth index, travel time to the nearest health facility, and literacy. Adjusting for those variables, the relationship between mobile phone ownership and ANC4+ was not statistically significant (aOR = 0.74; 95 % CI: 0.51–1.09; *p* = 0.133), nor was there a significant association between mobile phone ownership and skilled birth attendance (aOR = 1.29; 95 % CI: 0.67–2.51; *p* = 0.100) or health facility delivery (aOR = 1.13; 95 % CI: 0.56–2.28; *p* = 0.115). In multivariate regression for early postnatal care and early neonatal care, after including all the significant covariates identified in bivariate analysis, mobile phone ownership is removed from the regression model, with a *p* value larger than 0.20. No significant associations were found between mobile phone ownership and early neonatal and postnatal care.

## Discussion

Our findings demonstrate that women with household ownership of a mobile phone were more likely to utilize maternal and newborn health services. However, when adjusting for socioeconomic status, mobile phone ownership was not an independent predictor for antenatal care attendance, uptake of skilled birth attendance, health facility delivery, early postnatal or early neonatal care.

Women with a household mobile phone had higher socioeconomic status than those in homes without a phone. Mobile phone ownership may in fact serve as a surrogate for higher socioeconomic status, with low ownership in rural communities and among poor people [18, 20], and women with higher socioeconomic status are also more likely to utilize health services [30, 31]. Hence, women who had a mobile phone are *a priori* more likely to have greater access to health services. The 33 % of women in our study who did not have access to a mobile phone were less likely to utilize health services, and were thus most in need of the intervention.

Increasingly mHealth programs focus on the effects of interventions such as text messaging to disseminate health promotion information. Very few studies analyzed the limitations associated with unequal mobile phone access. Our findings indicate inequities related to socioeconomic status in use of maternal and newborn health services between mobile phone owners and those without a mobile phone. This is extremely important for the implementation of mHealth programs in low- or middle-income countries where unequal access to mobile phones still exists. As most mHealth programs focus on those with mobile phones, these findings support the concern that the populations without a mobile phone who are left out in the benefits of mHealth programs are actually the most in need of interventions. Even in some programs where women were given phone credit, they still could not fully benefit from the text messaging health education, or text reminders [11, 15]. Finally, supplying phones during mHealth programs or research studies is unlikely to be a sustainable approach once countries try to scale programs.

For maximum impact on the health of the general population, mHealth efforts should be integrated with other strategies to reach women who do not have access to mobile phones, such as household or community level health promotion. Strategies such as women’s groups, for example, could help those without a mobile phone access health information or contact health providers using the mobile phones of their group members. Collecting information on all women including those not enrolled in mHealth programs will help to better understand the populations not eligible for these efforts.

This study also suggests that the influence of mobile phones, even for women who have them, may not be sufficient to overcome the barriers to skilled delivery care that arise from structural factors such as lack of emergency transport, inadequate financial preparation for delivery, and deficiencies in quality of obstetric health services. In this study from Timor-Leste, mobile phone ownership was not independently associated with skilled birth attendance. One of the likely reasons for that finding is that emergency transport was not widely available in Timor-Leste. The power of mobile phones to help facilitate skilled birth attendance and health facility delivery may not be adequate to overcome greater barriers such as inadequate emergency obstetric transport, long distances from a facility, and other factors such as cultural or social influences. Hence, mHealth programs need to work in parallel with other interventions to improve the quality of care and to construct well-functioning emergency obstetric transport and referral systems.

### Limitations of the Study

This is the first study of which we are aware to address the inequity in maternal and newborn health services utilization among populations with and without access to a mobile phone. There are, however, limitations in our study. The survey on which the study is based did not involve questions related to the purposes of mobile phone usage, which makes it hard to explore whether these women had used their mobile phones to contact health providers, transport, or other women with experience of ANC or SBA. Another limitation lies in using mobile phone ownership as the independent variable, instead of mobile phone use. Among women in our study who reported having access to a mobile phone, only 82 % of the women reported it was their own phone, the rest using their family mobile phones. We did not examine these two groups independently and thus may not have accurately assessed the regular use of mobile phones by this population. The survey was conducted during the rainy season, and selection bias probably existed when researchers had to eliminate a few of the clusters in remote areas that were isolated by rivers and substitute some that were more accessible. In addition, interviewers did not check health care records to verify the reported care, and there was a higher level of postnatal and neonatal care reported in the survey than seen in other sources of district data.

Further studies would be useful to assess the effect of mobile phones on utilization of services, so as to better understand the mechanisms by which phone access may facilitate uptake. Additionally, qualitative research could investigate women’s views on how mobile phones could help them in contacting health providers, obtaining health knowledge related to pregnancy and childbirth, or building up social connection with women who have more experiences in delivery at health facilities or with skilled birth personnel. Further study would be helpful to assess the needs of the population that is not eligible for mHealth interventions and explore available methods to reach them.

## Conclusions

This study provides evidence that socioeconomic factors may be important barriers to the success of mHealth programs that focus exclusively on women having access to a mobile phone. Programs with an mHealth component will be most effective if they integrate with other interventions to also reach those who have limited or no access to mobile phones.

## Additional file

Additional file 1: English version of questionnaire. (DOCX 182 kb)

## Abbreviations

ANC: Antenatal Care
CATCH: Core Assessment Tool on Child Health
DHS: Demographic and Health Survey
HAI: Health Alliance International
KPC: Knowledge, Practices and Coverage
LMIC: Low and Middle Income Countries
MDG: Millennium Development Goal
mHealth: Mobile Health
MMR: Maternal Mortality Ratio
MNCH: Maternal, Newborn, and Child Health
NNC: Neonatal Care
PCA: Principal Component Analysis
PNC: Postnatal Care
SBA: Skilled Birth Attendants
SDG: Sustainable Development Goal
USAID: United States Agency for International Development
