# Supplementary material for: Does mobile phone ownership predict better utilization of maternal and newborn health services? a cross-sectional study in Timor-Leste
Source: BMC Pregnancy Childbirth. 2016 Jul 23;16:183. doi: 10.1186/s12884-016-0981-1 (PMC4958409; doi:10.1186/s12884-016-0981-1)
Supplement: Additional file 1: — English version of questionnaire. (DOCX 182 kb) [file 12884_2016_981_MOESM1_ESM.docx]

**Appendix 1— English version of questionnaire**

**Cover Page**

**Ask the mother if she has a child under 24 months who lives with her. If yes, proceed with interview, if no thank the mother and end the interview.**

| **Identification** | |  |
| --- | --- | --- |
| Record Number |  |  |
| Cluster Number |  |  |
| Household Number |  |  |
| Suco |  |  |
| Aldeia |  |  |
| Date of Interview | ___/___/_____  day/month/year |  |
| Language of interview | Tetun_____ Other (name)________________________ |  |
| Name of Interviewer |  |  |
| Name of Supervisor |  |  |
| Result Code* |  | |
| *Result Codes:   1. Completed 2. Respondent not at home 3. Refused 4. Other______________________________________   Specify | | |

| Data Entered by |  | Date: _____/_____/_____  day/month/year |
| --- | --- | --- |

**Consent Page**

Before beginning interview, ask if there is a woman in this household with a child under 2 years of age. If mother is between the ages of 15-17 and not yet married, a parent or legal guardian must be present during the consent stage and give their permission in order for the young woman to participate in this study. If the woman is older than 49 years of age, they may not participate in the study.

| **INFORMED CONSENT**  Hello. My name is ______________________________, and I am working with Health Alliance International and the Ministry of Health. We are conducting a survey and would appreciate your participation. I would like to ask you about your health and the health of your youngest child under the age of two. This information will help the Ministry of Health and Health Alliance International to plan health services and assess whether it is meeting its goals to improve maternal health. Is this a good time to talk?  The survey usually takes about 25 minutes to complete. Whatever information you provide will be kept strictly confidential and will not be shown to other persons. This data may be used again later by Health Alliance International or shared with the Ministry of Health, but your name will not be included.  Participation in this survey is voluntary and you can choose not to answer any individual question or all of the questions. However, we hope that you will participate in this survey since your views are important.  At this time, do you want to ask me anything about the survey?  Will you participate in this survey?  RESPONDENT AGREES TO BE INTERVIEWED _______ RESPONDENT DOES NOT AGREE TO BE INTERVIEWED _______  REQUIRED: If the woman is older than 15 years of age and under 17 years of age, and unmarried, does their parent or legal guardian give permission for her to participate in this survey?  PARENT OR LEGAL GUARDIAN GIVES PERMISSION FOR WOMAN TO BE INTERVIEWED _______  Signature of interviewer: _______________________________ Date: ____________________ |
| --- |

| NO. | QUESTIONS AND FILTERS | CODING CATEGORIES | SKIP |
| --- | --- | --- | --- |
| **Introduction** | | | |
| 1 | RECORD THE TIME | HOUR…………………..└───┴───┘  MINUTES………………└───┴───┘ |  |
| 2 | How old were you at your last birthday? | AGE IN COMPLETED YEARS |  |
| 3 | For how many years have you attended school?  IF NEVER, RECORD ‘00'. | YEARS IN SCHOOL |  |
| 4 | Do you work outside of the home to earn money?  IF NO, CIRCLE “A” (NO OUTSIDE WORK)  IF YES, What kind of work do you do? | NO OUTSIDE WORK 1  HANDICRAFTS 2  HARVESTING 3  SELLING FOODS 4  SHOP KEEPER/STREET VENDOR 5  SERVANT/HOUSEHOLD WORKER 6  SALARIED WORKER 7  OTHER 9  (SPECIFY) |  |
| 5 | Does your household have:  Electricity  A radio  A television  A mobile phone  A bicycle  A motorbike  A car or truck  Horse or other animal powered transport | ELECTRICITY 1  RADIO 2  TELEVISION 3  MOBILE PHONE 4  BICYCLE 5  MOTORBIKE 6  CAR OR TRUCK 7  HORSE OR ANIMAL-POWERED TRANSPORT 8 |  |
| 6 | OBSERVATION ONLY  MAIN MATERIAL OF THE ROOF. | PALM LEAVES 1  SHEET IRON 2  OTHER 9 |  |
| 7 | OBSERVATION ONLY  MAIN MATERIAL OF THE FLOOR. | EARTH 1  CONCRETE 2  WOODEN OR BAMBOO 3  OTHER 9 |  |
| 8 | How many children did you give birth to? | TOTAL NUMBER OF BIRTHS |  |
| 9 | How many children do you have that are living today? | TOTAL NUMBER OF CHILDREN |  |
| 10 | What is the name, sex, and month of birth of your youngest child that you gave birth to that is still alive?  COPY FROM THE LISIO/BIRTH BOOKLET IF AVAILABLE.  A) USED LISIO  B) USED RECALL/EVENTS CALENDER | Youngest Child  Name:  _______________________________ |  |
| **11** |  | **Sex**  **Male 1**  **Female 2** |  |
| 12 |  | Date of Birth    Month  Year |  |
| 13 | RECORD AGE OF CHILD IN MONTHS | MONTHS |  |
| 14 | IF THE NUMBER OF CHILDREN EVER BORN IS TWO OR MORE (CODE 2) | ONE CHILD EVER BORN (CODE 1) | **16** |
| 15 | What is the month of birth of your second youngest child that you gave birth to before (NAME)? | Second Youngest Child  Date of Birth  Month  Year |  |
| **Antenatal Care** | | | |
| 16 | During your pregnancy with (Name), did you see anyone for antenatal care?  IF YES: Whom did you see?  Anyone else?  PROBE FOR THE TYPE OF PERSON AND RECORD ALL PERSONS SEEN. | DOCTOR 1  NURSE 2  MIDWIFE 3  TRADITIONAL BIRTH ATTENDANT 4  OTHER 5  (SPECIFY)  NO ONE 9 | **21** |
| 17 | During your pregnancy with (Name), where did you receive antenatal care?  CIRCLE ALL MENTIONED.  IF SOURCE IS HOSPITAL, HEALTH CENTER, OR CLINIC, WRITE THE NAME OF THE PLACE. PROBE TO IDENTIFY THE TYPE OF SOURCE AND CIRCLE THE APPROPRIATE CODE.  _________________________________  (NAME OF PLACE) | HOME 1  HOSPITAL 2  MATERNITY………………………….....3  CHC 4  HEALTH POST 5  SISCa POST 6  OTHER OUTREACH EVENT 7  PRIVATE CLINIC 8  OTHER 9  (SPECIFY) |  |
| 18 | During your pregnancy with (Name), how many months pregnant were you when you first received antenatal care? | MONTHS  DON’T KNOW 9 |  |
| 19 | During your pregnancy with (Name), how many times did you receive antenatal care? | TIMES  DON’T KNOW 9 |  |
| 20 | As part of your antenatal care during this pregnancy, were any of the following done at least once?  A. Was your weight taken?  B. Was your blood pressure measured?  C. Did the midwife tell you your due date?  D. Did the midwife feel your stomach? | YES NO  A. WEIGHT 1 2  B. BP 1 2  C. DUE DATE 1 2  D. FEEL STOMACH 1 2 |  |
| 21 | During pregnancy, woman may encounter severe problems or illnesses and should go or be taken immediately to a health facility.  What types of symptoms would cause you to seek immediate care at a health facility (right away)?  ASK: Anything else?  DO NOT READ RESPONSES. RECORD ALL THAT ARE MENTIONED. | VAGINAL BLEEDING 1  FAST/DIFFICULT BREATHING 2  FEVER 3  SEVERE ABDOMINAL PAIN 4  HEADACHE/BLURRED VISION 5  CONVULSIONS 6  FOUL SMELLING DISCHARGE/FLUID FROM VAGINA 7  BABY STOPS MOVING 8  LEAKING BROWNISH/GREENISH FLUID FROM THE VAGINA .9  DOES NOT KNOW OF ANY…………..10  OTHER 19  (SPECIFY) | 23 |
| 22 | Where did you learn about these problems? | FROM A HEALTH CARE WORKER….1  IN LISIO…………………………………..2  ON A POSTER…………………………..3  CELL PHONE MESSAGE……………..4  RADIO MESSAGE…………………......5  IN A FILM OR VIDEO…………………..6  COMMUNITY HEALTH WORKER (PSF)……………………………………..7  OTHER _______________________ 8  (SPECIFY)  NA…………………………………………9 |  |
| 23 | During your pregnancy with (Name) did you receive an injection in the arm to prevent the baby from getting tetanus, that is convulsions after birth? | YES 1  NO 2  DON’T KNOW 9 | 25  25 |
| 24 | While pregnant with (name), how many times did you receive such an injection? | ONE 1  TWO 2  THREE OR MORE 3  DON’T KNOW 9 |  |
| 25 | Did you receive any tetanus toxoid injection at any time before that pregnancy, including during a previous pregnancy or between pregnancies? | YES 1  NO 2  DON’T KNOW 9 | 27  27 |
| 26 | Before the pregnancy with (Name), how many times did you receive a tetanus injection? | ONE 1  TWO 2  THREE OR MORE 3  DON’T KNOW 9 |  |
| 27 | During your pregnancy with (Name), were you given or did you buy any iron tablets?  SHOW TABLETS | YES 1  NO 2  DON’T KNOW 9 | 29  29 |
| 28 | During the whole pregnancy, for how many days did you take the tablets?  IF THE ANSWER IS NOT NUMERIC, PROBE FOR APPROXIMATE DAYS. | DAYS  DON’T KNOW 9 |  |
| 29 | During this pregnancy, did you take any drug for intestinal worms? | YES 1  NO 2  DON’T KNOW 9 |  |
| 30 | Did you sleep under a mosquito net during your pregnancy? | YES, ALL OR MOST OF THE TIME 1  YES, SOME OF THE TIME…………….2  NO 3  DON’T REMEMBER 9 |  |
| 31 | What kind of preparations did you make before the birth of (NAME)?  Anything else? RECORD ALL MENTIONED. | SAVED MONEY 1  BOUGHT CLEAN DELIVERY KIT 2  FOUND BLOOD DONOR 3  ARRANGED OF TRANSPORT 4  CONTACTED HEALTH WORKER TO HELP WITH DELIVERY 5  OTHER 8  (SPECIFY)  NO PREPARATION 9 |  |
| 32 | How long does it take you to travel by usual means to a facility where you could give birth? | MINUTES 0      HOURS 1 |  |
| 33 | What methods could you use to contact a midwife for assistance during delivery?  RECORD ALL MENTIONED. | IN PERSON ONLY 1  BY HOUSEHOLD PHONE 2  BY OTHER PHONE 3  SEND MESSENGER 4  OTHER 8  (SPECIFY)  NOT ABLE TO CONTACT MIDWIFE 9 |  |
| 34 | Where did you deliver (NAME)? | AT HOME 1  HOSPITAL 2  MATERNITY………………………….....3  CHC 4  HEALTH POST 5  PRIVATE CLINIC 6  OTHER 9 | 36 |
| 35 | What means of transportation did you use to travel to that facility? | WALK…………………………………….1  PRIVATE CAR…………………………..2  PUBLIC TRANSPORT (BUS)…………3  AMBULANCE OR MOH VEHICLE……4  OTHER (___________________)…….9 |  |
| 36 | Who assisted with the delivery of (NAME)?  Anyone else?  PROBE FOR THE TYPE(S) OF PERSON(S) AND RECORD ALL MENTIONED.  IF RESPONDENT SAYS NO ONE ASSISTED, PROBE TO DETERMINE WHETHER ANY ADULTS WERE PRESENT AT THE DELIVERY. | DOCTOR 1  NURSE 2  MIDWIFE 3  TRADITIONAL BIRTH ATTENDANT 4  PSF 5  HUSBAND………………………………..6  OTHER RELATIVE/FRIEND 7  NO ONE 8 |  |
| 37 | What instrument was used to cut the cord? | NEW RAZOR BLADE 1  NEW AND BOILED RAZOR BLADE 2  USED RAZOR BLADE 3  USED AND BOILED RAZOR BLADE 4  SCISSORS 7  BOILED SCISSORS 8  KNIFE 9  BAMBOO OR NATURAL MATERIAL 10  OTHER 18  (SPECIFY)  DON’T KNOW 19 |  |
| 38 | Was anything placed on the umbilical cord either before or after it was cut? | YES 1  NO 2  DON’T KNOW 9 | 40  40 |
| 39 | What was placed on the cut cord? | COW DUNG 1  ANY TYPE OF OIL 2  ANTISPETIC 3  ASH 4  OTHER (SPECIFY) 9 |  |
| 40 | Was (NAME) wiped dry immediately after birth, before the placenta was delivered? | YES 1  NO 2  DON’T KNOW 9 |  |
| 41 | Was (NAME) wrapped in a cloth or blanket immediately after birth, before the placenta was delivered? | YES 1  NO 2  DON’T KNOW 9 |  |
| 42 | Immediately after (NAME) was born, before the placenta was delivered, did you receive an injection to prevent you from bleeding too much? | YES 1  NO 2  DON’T KNOW 9 |  |
| 43 | Immediately after the Placenta was delivered, did someone massage your uterus to make it contract strongly and to prevent you from bleeding too much? | YES 1  NO 2  DON’T KNOW 9 |  |
| 44 | During delivery, once contractions started, a woman may encounter severe problems or illnesses and should go or be taken immediately to a health facility.  While having contractions or delivering a baby, what types of symptoms would cause you to seek immediate care at a health facility (right away)?  ASK: Anything else?  DO NOT READ RESPONSES. RECORD ALL THAT ARE MENTIONED. | CONVULSIONS 1  HIGH FEVER 2  HEAVY BLEEDING 3  FAST/DIFFICULT BREATHING 4  RETAINED PLACENTA 5  HEADACHE/BLURRED VISION 6  PROLONGED LABOUR 7  DON’T KNOW 8  OTHER 9  (SPECIFY) |  |
| 45 | Did you ever breastfeed (NAME)? | YES 1  NO 2 | 51 |
| 46 | How long after birth did you first put (NAME) to the breast?  IF LESS THAN 1 HOUR, RECORD 00 HOURS,  IF LESS THAN 24 HOURS RECORD THE HOURS,  OTHERWISE RECORD DAYS | IMMEDIATE 00  HOURS  DAYS  DON’T REMEMBER 9 |  |
| 47 | Before you began breastfeeding (NAME), was he/she given anything to drink? | YES 1  NO 2  DON’T KNOW 9 | 49  49 |
| 48 | What else was (NAME) given to drink?  Anything else?  Record all liquids mentioned. | PLAIN WATER 1  SUGAR OR GLUCOSE WATER 2  PORRIDGE/GRUEL (SOSORRO) 3  INFANT FORMULA 4  OTHER BREAST MILK  (FAMILY/NEIGHBOUR) 5  MILK (OTHER THAN BREAST MILK) 6  OTHER 9 |  |
| 49 | Did you give the baby the first liquid (Colostrum) that came from your breasts? | YES 1  NO 2  DON’T KNOW 9 |  |
| 50 | In the first month after delivery, was (NAME) given anything to drink other than breast milk? | YES 1  NO 2  DON’T KNOW 9 |  |
| 51 | In the first hour after delivery, was (NAME) given eye ointment or drops in his/her eyes? | YES 1  NO 2  DON’T KNOW 9 |  |
| **Postpartum/Postnatal Care** | | | |
| 52 | Did a health worker check on your health after the delivery of (NAME), either at a health facility, home or other location? | YES 1  NO 2 | 55 |
| 53 | How long after the delivery did the first check take place?  IF LESS THAN ONE DAY, CIRCLE 0 AND RECORD HOURS; IF LESS THAN ONE WEEK CIRCLE 1 AND RECORD DAYS; IF MORE THAN 6 DAYS CIRCLE 2 AND RECORD WEEKS. | HOURS 0      DAYS 1  WEEKS 2    DON’T KNOW 9 |  |
| 54 | Who checked your health at that time?  Anyone else?  PROBE FOR THE MOST QUALIFIED PERSON AND RECORD ALL MENTIONED. | DOCTOR 1  NURSE 2  MIDWIFE 3  TRADITIONAL BIRTH ATTENDANT 4  PSF 5 |  |
| 55 | Sometimes mothers after delivery have severe illnesses and should be taken immediately to a health facility.  What types of symptoms would cause you to go to a health facility right away?  ASK: Anything else?  DO NOT READ RESPONSES. RECORD ALL THAT ARE MENTIONED. | EXCESSIVE VAGINAL BLEEDING 1  FAST/DIFFICULT BREATHING 2  HIGH FEVER 3  SEVERE ABDOMINAL PAIN 4  SEVERE HEADACHE/BLURRED VISION 5  CONVULSIONS/LOSS OF CONSCIOUSNESS 6  FOUL-SMELLING DISCHARGE FROM THE VAGINA 7  PAIN IN CALF 8  VERBALIZATION/BEHAVIOR THAT INDICATES SHE MAY HURT HERSELF OR THE BABY 9  DON’T KNOW 10  OTHER 20  (SPECIFY) |  |
| **The following questions refer to the youngest child shortly after birth** | | | |
| 56 | After (NAME) was born, did any health care provider or traditional birth attendant check on (NAME’s) health? | YES 1  NO 2 | 59 |
| 57 | How many hours, days or weeks after the birth of (Name) did the first check take place?  IF LESS THAN ONE DAY, CIRCLE 0 AND RECORD HOURS; IF ONE TO SIX DAYS CIRCLE 1 AND RECORD DAYS; IF MORE THAN 6 DAYS CIRCLE 2 AND RECORD WEEKS. | HOURS 0      DAYS 1  WEEKS 2    DON’T KNOW 9 |  |
| 58 | Who checked on (Name’s) health at that time?  Anyone else?  PROBE FOR THE MOST QUALIFIED PERSON AND RECORD ALL MENTIONED. | DOCTOR 1  NURSE 2  MIDWIFE 3  TRADITIONAL BIRTH ATTENDANT 4  PSF 5  RELATIVE/FRIEND 6 |  |
| 59 | Sometimes newborns, within the first month of life, have severe illnesses and should be taken immediately to a health facility.  What types of symptoms would cause you to take your newborn to a health facility right away?  ASK: Anything else?  DO NOT READ RESPONSES. RECORD ALL THAT ARE MENTIONED. | CONVULSIONS 1  FEVER 2  POOR SUCKLING OR FEEDING 3  FAST/DIFFICULT BREATHING……… 4  BABY FEELS COLD 5  BABY TOO SMALL/TOO EARLY 6  YELLOW PALMS/SOLES/EYES 7  SWOLLEN ABDOMEN 8  UNCONSCIOUS 9  PUS OR REDNESS OF THE UMBILICAL STUMP, EYES OR SKIN 10  DON’T KNOW 11  OTHER 19  (SPECIFY) |  |
| 60 | How long should you wait after the birth of your child before you try to become pregnant again?  DO NOT READ RESPONSES | 1 YEAR OR LESS………………………1  2 YEARS 2  3 TO 5 YEARS 3  MORE THAN 5 YEARS 4  DON’T KNOW 9 |  |
| 61 | What are the risks of getting pregnant too soon after the birth of a child?  DO NOT READ RESPONSES. RECORD ALL THAT ARE MENTIONED. IF ‘BAD FOR HEALTH’ IS THE ANSWER, ASK FOR MORE SPECIFIC RESPONSE (“HOW IS IT BAD?”) | BABY BORN TOO SMALL 1  BABY BORN TOO EARLY 2  MOTHER CAN DIE 3  MOTHER CAN HAVE MISCARRIAGE 4  MOTHER CAN SUFFER ANEMIA 5  BAD FOR HEALTH OF MOTHER  AND/OR BABY……………………..……6  DON’T KNOW……………………..……7  OTHER 9  (SPECIFY) |  |
| 62 | Can you name some methods that could be used to space your childrens’ births, meaning contraceptive methods?  CIRCLE ALL METHODS THAT ARE SPONTANEOUSLY NAMED    AFTER EACH RESPONSE PROBE FOR ‘ANY OTHERS?’ | FEMALE STERILIZATION 1  MALE STERILIZATION 2  PILL 3  IUD 4  INJECTABLE (DEPO PROVERA) 5  IMPLANTS 6  CONDOMS 7  LACTATIONAL AMEN. METHOD 8  STANDARD DAYS METHOD/ CYCLEBEADS 9  RHYTHM METHOD (OTHER  THAN STANDARD DAYS) 10  DON’T KNOW……………………..……11  OTHER 13 |  |
| 63 | Have you ever heard of these other methods of contraception?  READ OFF EACH METHOD THAT THE RESPONDENT DID NOT ALREADY LIST, AND CIRCLE THE NUMBER IF SHE SAYS SHE HAS HEARD OF IT. | FEMALE STERILIZATION 1  MALE STERILIZATION 2  PILL 3  IUD 4  INJECTABLE (DEPO PROVERA) 5  IMPLANTS 6  CONDOMS 7  LACTATIONAL AMEN. METHOD 8  STANDARD DAYS METHOD/ CYCLEBEADS 9  RHYTHM METHOD (OTHER  THAN STANDARD DAYS) 10  DON’T KNOW……………………..……11  OTHER 13 |  |
| 64 | Are you currently doing something or using any method to delay or avoid getting pregnant? | YES 1  NO 2 | 66 |
| 65 | Which method are you (or your husband/ partner) using?  DO NOT READ RESPONSES. CODE ONLY ONE RESPONSE.  IF MORE THAN ONE METHOD IS MENTIONED, ASK,  What is your MAIN method that you (or your husband/ partner) use to delay or avoid getting pregnant?”  IF REPONDENT MENTIONS BOTH CONDOMS AND STANDARD DAYS METHOD, CODE “12” FOR STANDARD DAYS METHOD  IF RESPONDENT MENTIONS ABSTINENCE OR ISOLATION, CODE “13” FOR OTHER AND SPECIFY RESPONSE IN SPACE PROVIDED.  IF RESPONDENT MENTIONS BREASTFEEDING ONLY, CODE AS BREASTFEEDING, NOT LAM | FEMALE STERILIZATION 1  MALE STERILIZATION 2  PILL 3  IUD 4  INJECTABLE (DEPO PROVERA) 5  IMPLANTS 6  CONDOMS 7  LACTATIONAL AMEN. METHOD 8  STANDARD DAYS METHOD/ CYCLEBEADS 9  RHYTHM METHOD (OTHER  THAN STANDARD DAYS) 10  BREASTFEEDING………………………11  WITHDRAWAL 12  OTHER 13 (SPECIFY) |  |
| **Literacy** | | | |
| 66 | Can you speak, read, or write Tetun Prasa? | CANNOT SPEAK, READ, OR WRITE 1  SPEAK ONLY 2  SPEAK AND READ ONLY 3  SPEAK, READ, AND WRITE 4 | **68**  **68** |
| 67 | If you cannot read Tetun, is there someone in your household or nearby who can read you a simple message in Tetun? | HUSBAND 1  CHILD 2  OTHER FAMILY MEMBER 3  NEIGHBOR 4  PSF 5  OTHER 9  (SPECIFY) |  |
| **Cell Phone Ownership and Questions** | | | |
| 68 | Do you or any of your household members own a mobile phone? | YES 1  NO 2 | **79** |
| 69 | How many mobile phones are there in your household? | NUMBER OF PHONES |  |
| 70 | Do you receive regular cell phone signal within your house or must you walk to another location? | HAVE SIGNAL IN HOME 1  WALK LESS THAN 5 MINUTES 2  WALK MORE THAN 5 MINUTES 3  THERE IS NO SIGNAL IN  COMMUNITY 4 |  |
| 71 | How do you charge the phone?  RECORD ONLY MOST FREQUENT METHOD USED. | ELECTRICTY AT HOME 1  THROUGH BATTERY AT HOME 2  ELECTRICITY AT OTHER SITE 3  OTHER 9  (SPECIFY) |  |
| 72 | Do you own your own phone or do you regularly use one of the family phones? | OWN PHONE 1  USES FAMILY PHONE 2  DOES NOT USE PHONE 2 | **7** |
| 73 | How often do you use the cell phone? | MULTIPLE TIMES A DAY 1  ONCE A DAY 2  ONCE A WEEK 3  ONCE A MONTH 4  LESS FREQUENTLY 5  NOT AT ALL……………………………..6 |  |
| 74 | How often is the phone you can use turned on? | ALL THE TIME 1  AT LEAST ONCE A DAY 2  AT LEAST ONCE A WEEK 3  LESS FREQUENTLY 4 |  |
| 75 | Do you use the phone to receive or send text messages? | YES 1  NO 2 |  |
| 76 | How often do you send text messages from the phone? | MULTIPLE TIMES A DAY 1  ONCE A DAY 2  ONCE A WEEK 3  ONCE A MONTH OR LESS 4  NEVER 5 |  |
| 77 | We are investigating a program that could send health messages to women during pregnancy.  If you were enrolled in this program, in which language would you prefer to receive health messages on your cell phone? | TETUN PRASA 1  BAHASA INDONESIAN 2  PORTUGUESE 3  MAMBAI 4  ENGLISH 5  OTHER 9  (SPECIFY) |  |
| 78 | What time of day is best for receiving text messages? | MORNING 1  AFTERNOON 2  EVENING 3  NO PREFERENCE 4 |  |
| 79 | RECORD THE TIME | HOUR…………………..└───┴───┘  MINUTES………………└───┴───┘ |  |

Thank you so much for talking to us. We appreciate that you have shared some important experiences. Your answers will be combined with those from other mothers in this *suco* and throughout Timor-Leste, and will really help us to find better ways to improve the health of mothers and children.
